# Development and Characterization of an Anti-Cancer Monoclonal Antibody for Treatment of Human Carcinomas

**DOI:** 10.3390/cancers14133037

**Published:** 2022-06-21

**Authors:** Kwong yok Tsang, Massimo Fantini, Sharon A. Mavroukakis, Anjum Zaki, Christina M. Annunziata, Philip M. Arlen

**Affiliations:** 1 Precision Biologics, Inc., Bethesda, MD 20814, USA; massimo.fantini@precision-biologics.com (M.F.); sharon.mavroukakis@precision-biologics.com (S.A.M.); anjum.zaki@precision-biologics.com (A.Z.); philip.arlen@precision-biologics.com (P.M.A.); 2 Women’s Malignancy Branch, Center for Cancer Research, National Cancer Institute, National Institutes of Health, Bethesda, MD 20892, USA; annunzic@mail.nih.gov

**Keywords:** NEO-201, monoclonal antibody, tumor-associated antigen, antibody-dependent cellular cytotoxicity, complement-dependent cytotoxicity, natural killer cells

## Abstract

**Simple Summary:**

Cancers can grow and spread to different parts of the body. The aim of immunotherapy is to stimulate the immune system to eliminate cancer cells in a selective manner. Tumor-targeting monoclonal antibodies (mAb) can be used as immunotherapy to stimulate innate antitumor immunity. In this review, we have described the development and characterization of an anti-cancers mAb NEO-201. NEO-201 is an IgG1 humanized mAb that binds specifically to tumor-associated variants of CEACAM-5 and CEACAM-6 expressed by colon, ovarian, pancreatic, non-small cell lung, head and neck, cervical, uterine and breast cancers, but is not reactive against most normal tissues. The peculiarity of NEO-201 is its ability to counteract tumor growth through different mechanisms. NEO-201 engages components of immune system to kill tumor cells expressing its target via antibody-dependent cell-mediated cytotoxicity and complement dependent cytotoxicity. NEO-201 can also indirectly enhance anti-cancer activity through the blockade of the interaction between CEACAM-5 expressed on tumor cells and CEACAM-1 expressed on natural killer cells to reverse CEACAM-1-dependent inhibition of NK cytotoxicity or through its binding to human regulatory T cells. The specificity of NEO-201 in recognizing suppressive regulatory T cells provides the basis for combination cancer immunotherapy with checkpoint inhibitors targeting the PD-1/PD-L1 pathway.

**Abstract:**

NEO-201 is an IgG1 humanized monoclonal antibody (mAb) that binds to tumor-associated variants of carcinoembryonic antigen-related cell adhesion molecule (CEACAM)-5 and CEACAM-6. NEO-201 reacts to colon, ovarian, pancreatic, non-small cell lung, head and neck, cervical, uterine and breast cancers, but is not reactive against most normal tissues. NEO-201 can kill tumor cells via antibody-dependent cell-mediated cytotoxicity (ADCC) and complement dependent cytotoxicity (CDC) to directly kill tumor cells expressing its target. We explored indirect mechanisms of its action that may enhance immune tumor killing. NEO-201 can block the interaction between CEACAM-5 expressed on tumor cells and CEACAM-1 expressed on natural killer (NK) cells to reverse CEACAM-1-dependent inhibition of NK cytotoxicity. Previous studies have demonstrated safety/tolerability in non-human primates, and in a first in human phase 1 clinical trial at the National Cancer Institute (NCI). In addition, preclinical studies have demonstrated that NEO-201 can bind to human regulatory T (Treg) cells. The specificity of NEO-201 in recognizing suppressive Treg cells provides the basis for combination cancer immunotherapy with checkpoint inhibitors targeting the PD-1/PD-L1 pathway.

## 1. Introduction

The immune system recognizes and suppresses the proliferation of transformed malignant cells through the mechanism of immunoediting. The immunoediting process is composed of three phases: elimination, equilibrium, and escape [1]. During the elimination phase, normal cells are transformed in cancer cells through a multistep process called carcinogenesis. This process is due to the upregulation of the expression of oncogenes (genes that induce cell proliferation), and/or to the downregulation of the expression or loss of function of onco-suppressor genes (genes involved on the control of cell growth). As a result, transformed cancer cells acquire the ability to proliferate in an uncontrolled manner and become unresponsive to negative regulators of growth and survival [1,2].

During the elimination phase, both the innate and adaptive immune system work together to recognize and eliminate tumors at an early stage before they become clinically visible [1]. NK cells, macrophages, granulocytes (components of innate immune system), and cytotoxic T lymphocytes (CTLs) (a component of adaptive immune system) recognize and bind to antigens expressed on the surface of cancer cells. After the binding, immune system cells eliminate tumor cells through different mechanisms. For example, NK cells and CTLs can induce tumor cell apoptosis or can directly lyse tumor cells by secreting perforin and granzymes [1,3].

In the equilibrium phase, the immune system maintains the tumor in a condition of dormancy [1].

In the escape phase, cancer cells evade the immune system pressure, acquiring a more aggressive phenotype and the ability to grow without control and to spread to different parts of the body from the original tumor site [1,2]. Mechanisms of evasion from immune recognition include loss of MHC class I molecules, expression of neo-antigens, upregulation of anti-apoptotic genes, over-expression of immunosuppressive molecules (IDO, PD-L1), increased secretion of cytokines that enhance angiogenesis (VEGF, TGF-β, IL-6, M-CSF). Furthermore, tumor cells can secrete colony stimulating factors (G-CSF and GM-CSF) leading to the accumulation of myeloid-derived suppressor cells (MDSCs) in the tumor microenvironment (TME). These cells release immunosuppressive cytokines, such as IL-10 and TGF-β, leading to the exhaustion of CTLs. MDSCs can also induce the generation of regulatory T cells. Regulatory T cells are able to suppress anti-tumor immune response through the expression of inhibitory receptors and cytokines (TGF-β, IL-10, IDO, Tim-3 and LAG-3) [1,4,5,6,7].

Cancer immunotherapy can play a fundamental role in preventing the escape phase of immunoediting, enhancing the pressure of the immune system to keep cancer cells constantly in the elimination and/or equilibrium phase [8]. Several cancer immunotherapies may have the potential to keep cancer cells in the elimination and/or equilibrium phase. These include check-point inhibitor antibodies, anticancer vaccines, peptide vaccines, and chimeric antigen receptor-T cells (CAR-T cells), all of which leverage adaptive immunity by T cells [4].

Tumor-targeting monoclonal antibodies (mAbs) can be used to stimulate innate antitumor immunity.

One of the mechanisms used by tumor cells to evade immunosurveillance is the activation of immune checkpoint pathways that allow an uncontrolled tumor growth through the suppression of antitumor immune responses. The employment of FDA-approved monoclonal antibodies, that inhibit immune checkpoints (immune checkpoints inhibitors, ICIs) and enhance immune anti-tumor responses to eliminate tumor cells, represents a big innovation in cancer immunotherapy [9].

The first FDA-approved monoclonal antibody used as checkpoint inhibitor was ipilimumab, which targets cytotoxic T lymphocyte antigen-4 (CTLA-4), a molecule expressed by activated T-cells. The mechanism of action of this antibody is to bind to CTLA-4, interfering with immune inhibitory signals generated through this molecule, thus preventing inhibition of T-cell function and promoting T-cell activation and proliferation [10].

Other important pathways targeted by anti-cancer monoclonal antibodies are the epidermal growth factor receptor (EGFR)-, CD20-, vascular endothelial growth factor (VEGF)-, and the programmed cell death protein-1 (PD-1)/programmed cell death protein-1 ligand (PD-L1)-mediated pathways [11].

Although immunotherapy with monoclonal antibodies has increased survival of cancer patients, a significant portion of patients fail to respond to therapy. This phenomenon is due in part to both primary and acquired resistance to ICIs [12].

For example, low expression of tumor antigens, uncontrolled upregulation of oncogenes, increased activity of regulatory T cells and myeloid-derived suppressor cells to impair immune system in the tumor microenvironment can lead to the resistance to ICIs [13].

To overcome this issue, monoclonal antibodies were engineered to have different mechanisms of action, including ADCC [14], CDC [15,16,17], antibody-dependent cellular phagocytosis (ADCP) [18,19], receptor blocking [20] and ligand blocking [21,22], as well as bispecific T-cell engager (BiTE) [23,24] and antibody drug conjugates (ADC) [25,26].

Examples of clinically approved mAbs that can mediate ADCC include trastuzumab, rituximab, cetuximab and avelumab [27].

NEO-201 is an IgG1 humanized mAb targeting a variant of CEACAM-5 and CEACAM-6, members of the carcinoembryonic antigen (CEA) family of proteins. NEO-201 is reactive against many different carcinomas, but not reactive against most normal tissues. Functional analysis revealed that NEO-201 can engage innate immune effector mechanism including ADCC and CDC to directly kill tumor cells expressing its target [28,29,30]. We explored indirect mechanisms of its action that may enhance immune tumor killing. NEO-201 can block the interaction between CEACAM-5 expressed on tumor cells and CEACAM-1 expressed on NK cells to reverse CEACAM-1-dependent inhibition of NK cytotoxicity [31]. We also showed that NEO-201 can bind to human regulatory T cells in peripheral blood mononuclear cells (PBMCs) from healthy donors in vitro [32]. An open label, first in human phase 1 dose escalation study was recently completed at the NCI. This review focuses on this anti-cancer agent NEO-201 with respect to the development, characterization, known mechanisms of action, current clinical applications for treatment of cancers.

## 2. Generation of mAb NEO-201

**Figure 1:**
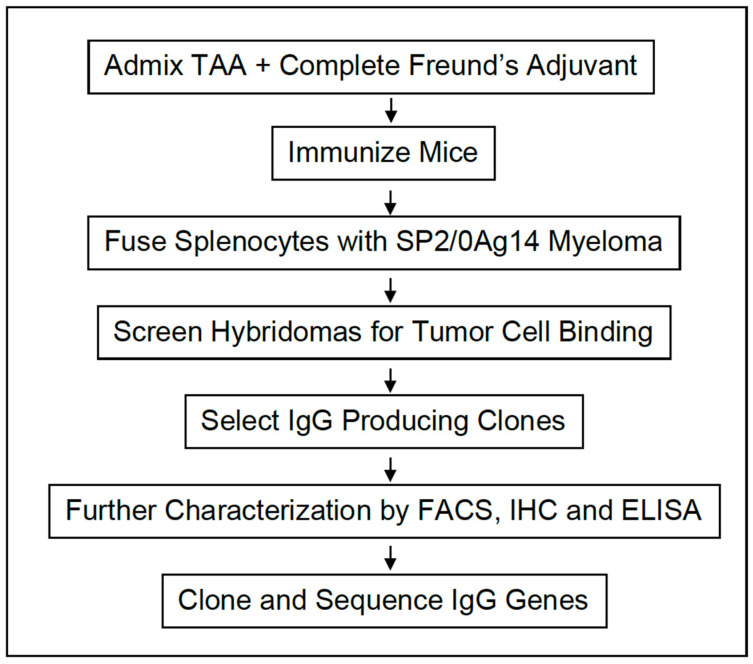
Flow diagram of monoclonal antibody production.

The murine monoclonal antibody 16C3 was developed against an immunogenic tumor-associated antigen (TAA) using pooled allogeneic colon tumor tissue extracts described by Hollinshead [28,33,34,35]. Figure 1 describes the production of the murine mAb 16C3. The TAA consisted of two stable polypeptides with approximate molecular weights of 72 kDa and 88 kDa, respectively, as compared with the 180 kDa molecular weight of CEA [33,34].

The murine 16C3 clone E12 was chosen for further development based on the strong binding to colon cancer cell lines LS174T and HT29 as analyzed by ELISA. The m16C3 clone E12 protein sequence was humanized as h16C3 and designated NEO-201 [28].

## 3. Identification of Binding Targets by Flow Cytometry

A panel of human carcinoma cell lines was used to analyze the binding activity of NEO-201 using flow cytometry. NEO-201 was found to bind to a variety of human carcinoma cell lines in vitro, including colon, pancreatic, lung and breast cancer cells.

In particular, NEO-201 positivity was observed in 50% of the colon cancer cell lines and 80% pancreatic cancer cell lines tested. In Non-Small Cell Lung Cancer (NSCLC) cells, reactivity with NEO-201 was detected more frequently in tumor cell lines derived from lung adenocarcinomas versus squamous cell carcinomas.

NEO-201 was also observed to bind to 50% of breast cancer cell lines expressing either the estrogen receptor (ER) or the progesterone receptor (PR), whether alone or in combination with HER2 and to 75% of the HER2+ cell lines. On the contrary, NEO-201 recognized only 25% of triple-negative breast cancer cell lines tested [28].

## 4. Identification of Binding Targets by Immunohistochemistry (IHC)

To identify the specific antigen targeted by NEO-201 on tumor cells, immunoprecipitation of NEO-201 target antigen was performed comparing different human cancer cells lines highly reactive for NEO-201 with cells lines that do not react with NEO-201. From these screening CEACAM-5 and CEACAM-6 were identified as the most likely targets of NEO-201 [29].

To confirm that NEO-201 binds specifically to cancer cells expressing tumor-associated variants of CEACAM-5 and CEACAM-6, which are not expressed in normal tissues, we used human tissue microarrays of various cancer types for immunohistochemistry analysis. The reactivity of NEO-201 on colon, pancreas, lung normal and cancer tissues was compared to the reactivity of commercially available anti-CEACAM-5 and CEACAM-6 mAbs.

**Figure 2:**
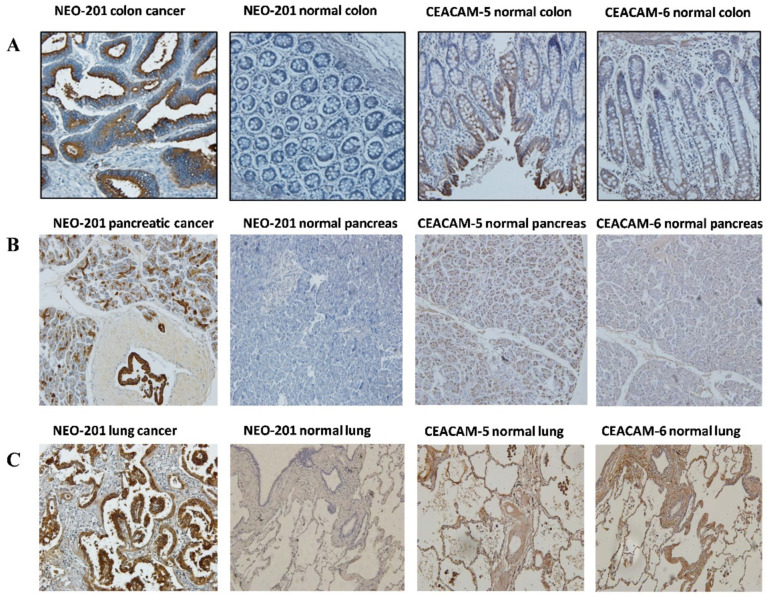
NEO-201 IHC of tumor tissue microarrays. Immunohistochemistry peroxidase staining of NEO-201 mAb (h16C3) and commercial CEACAM 5/6 antibodies in (**A**) colon cancer and normal colon tissue, (**B**) pancreatic cancer and normal pancreas tissue, and (**C**) lung cancer and normal lung tissue. All images were obtained at 100X.

Using 32 different colon adenocarcinoma tissue samples, 28/32 (87.5%) were found to stain positive for NEO-201, while 0/32 positivity was seen in the normal adjacent colon tissue. In comparison, commercially available anti-CEACAM-5 mAb stained positive in 31/32 (96.9%) colon cancer specimens and 29/32 (90.6%) of adjacent normal tissue. Commercially available anti-CEACAM-6 mAb stained positive in 31/32 (96.9%) of colon cancer specimens and 29/32 (93.6%) of adjacent normal tissue. This is consistent for pancreatic cancer and lung cancer and with data previously published [28] (Figure 2, Table 1). These data confirmed that NEO-201 reactivity is tumor specific in epithelial cells.

In addition, as shown in Table 1, NEO-201 has also been found to react against colon, pancreatic, stomach, lung, breast, and ovarian cancer tissues. The strongest reactivity was observed from the pancreatic and colon cancer samples (Figure 2 and Table 1).

In a previous study, we further evaluated NEO-201 binding activity in the tissues of patients with different ovarian cancer subtypes, using a tissue microarray (TMA) containing 627 ovarian cancer samples, including 11 ovarian cancer histological subtypes. Interestingly, we observed that mucinous adenocarcinoma showed the highest percentage of positive (68%) samples among all the histological subtypes analyzed, while only 20% of serous adenocarcinoma and 38% of germinal cell tumors were positive for NEO-201 staining [29].

When we further investigated the reactivity of NEO-201 to lung cancer, using a panel of 75 lung cancer tissues of different histotypes, we observed that NEO-201 strongly reacted to lung adenocarcinoma tissues (79.4%), while only 52.9% of lung squamous cell cancer tissues stained positive for NEO-201 (Table 2).

## 5. NEO-201 Can Also Target Human Acute Myeloid Leukemia (AML) and Multiple Myeloma (MM) Cell Lines In Vitro

AML is a neoplasm of immature myeloid cells and is associated with a wide variety of clinical presentations, morphological features, immunophenotypes, and genetic findings. Recent advances in identification of cytogenetic abnormalities and mutations have provided novel insights into the pathogenesis of AML. Based on the above-mentioned parameters, the World Health Organization (WHO) classified AML into 25 subtypes, including two provisional entities, which differ in prognosis and treatment [36]. Despite major progresses in AML therapy, many patients still relapse and die from the disease. Unfortunately, there is no curative therapy for patients who relapse, except for a small proportion who are cured by allogeneic stem cell transplant (allo-SCT) [37].

Only the anti-CD33 antibody drug conjugate gemtuzumab ozogamicin is currently approved by the FDA as an antibody-targeted therapy for AML [38].

MM is the second most common hematological malignancy, accounting for 13% of blood cancers, and is characterized by an aberrant proliferation of plasma cells (PCs) that enhance the production of monoclonal immunoglobulins. The accumulation of abnormal monoclonal immunoglobulins impairs the body’s immune response against pathogens, making it susceptible to infections. In the last few decades several effective treatments have been developed against MM, including target-specific monoclonal antibodies, ADCs, and bispecific Abs. However, toxicity still remains a concern for these treatment strategies [39].

In a previous study, flow cytometry analysis of human hematopoietic cells for NEO-201 binding revealed that 98.9% of CD15^+^ granulocytes and about 4.6% of CD4^+^ T cells were reactive with NEO-201. No reaction with NEO-201 was observed in B cells, NK cells, monocytes, CD8^+^ T cells and a majority of CD4^+^ T cells [31].

In addition, we also demonstrated that the CD4^+^/NEO-201^+^ subset in human PBMCs from healthy donors had the regulatory T cells phenotype [32].

Since NEO-201 target antigen is expressed both on solid tumors as well as in CD15^+^ granulocytes and regulatory T cells, we evaluated the reactivity of NEO-201 against hematological neoplastic cell lines in vitro, such as Acute Myeloid Leukemia (AML), Multiple Myeloma (MM), Acute Lymphoblastic Leukemia (ALL), Mantel Cell Lymphoma (MCL) cells in flow cytometry. NEO-201 was found to react with 5 AML and 2 MM cell lines. Five of six AML cell lines tested were highly positive for NEO-201. The percentage of positive cells in the two MM cell lines tested were 99% and 18% for OPM2 and MM1.S, respectively. NEO-201 did not react against the two ALL and the four MCL cell lines tested. We also observed that NEO-201 was able to kill the AML cell line HL60 via ADCC [40].

These findings provide a rationale for further investigation of the role of NEO-201 as a therapeutic agent to treat AML as well as MM.

## 6. Mechanisms of Action of NEO-201

Anti-cancer mAbs possess a variety of mechanisms in directing cytotoxic effects to cancer cells. The direct mechanism by which mAbs induce tumor cell death includes the blockade of growth factor receptor signaling, (anti-EGFR mAbs), or blocking ligand binding and receptor dimerization (anti-EGFR/HER2 heterodimerization and activation) [22]. Most anti-cancer mAbs can also kill tumor cells, interacting with components of the immune system, through ADCC, CDC or ADCP [14,15,16,17,18,19].

### 6.1. NEO-201-Mediated ADCC and CDC against Human Tumor Cells

As previously noted, NEO-201 binds to a wide range of human carcinoma cells and tissues. Prior studies demonstrated that NEO-201 is able to mediate ADCC against cancer cells expressing high levels of its target antigen, including human pancreatic (CFPAC-1 and ASPC-1), breast (ZR-75-1), lung (H520 and HCC827) and ovarian (OV-90) cancer cell lines [28,29,30]. In addition, we have previously shown that NEO-201 mediates CDC to kill the pancreatic cancer cell line ASCP-1 in vitro [28]. mAbs that mediate ADCC and/or CDC may play a role to improve the clinical response of cancer patients.

ADCC is a mechanism occurring when the fragment crystallizable (Fc) region of mAbs binds to the Fc gamma receptor IIIa (FcƴRIIIa, CD16) expressed on macrophages and NK cells. This interaction activates macrophages to phagocytose mAb-opsonized cancer cells and induces NK cells to kill mAb-bounded cancer cells [14].

Additionally, the Fc region can also interact with the C1 complex, activating a proteolytic cascade that leads to the formation of pores in the plasma membrane of opsonized target cells and the consequent lysis of tumor cells targeted by the antibody. This mechanism is known as CDC [15].

The advantage of using mAbs able to trigger both ADCC and CDC is that they not only activate NK cells, but also stimulate the secretion of IFN-γ, TNF-α, and other chemokines which in turn recruit immune effector cells to the tumor site. In this manner, these mAbs have the capability to counteract tumor growth through an increase in tumor antigens presentation to effector cells and direct lysis of cancer cells [14,15].

Importantly, antitumor efficacy of CDC has been demonstrated in vitro, but the contribution of complement to the clinical efficacy of mAbs employed in cancer immunotherapy remains controversial [15,41]. This phenomenon could be due to the expression of several different complement-regulatory proteins (CRPs) by tumor cells, such as CD46, CD55, and CD59, which inhibit complement activation and confer resistance of tumor cells to killing mediated by CDC [42]. Future investigations will ascertain whether strategies to block CRPs can enhance NEO-201-mediated CDC of resistant tumor cells.

### 6.2. ADCC Activity Medited by NEO-201 Can Be Enhanced by IL-15

Cytokines such as IL-15 can enhance the anti-cancer activity of NK cells. IL-15 can modulate NK cell development, proliferation, cytotoxicity, and cytokine production [30]. IL-15 binds to IL-15Rα present on the surface of immune cell subsets such as monocytes, dendritic cells, NK cells and CD8^+^ T cells where it forms a complex with IL-15Rβ to activate different intracellular signaling pathways, including the PI3K-AKT-mTOR pathway [43,44].

Despite promising results in the employment of IL-15 as a cytokine to boost anti-tumor immune responses, its efficacy is limited by the short in vivo half-life and by the availability of IL-15Rα [45]. To overcome this issue, novel engineered IL-15 molecules have been developed, including the IL-15 superagonist complex N-803 (formerly known as ALT-803).

N-803 consists of an IL-15 variant (IL-15N72D) bound to an IL-15 receptorα/IgG1 FC fusion protein. This complex has been proven to have improved stability, longer persistence in lymphoid tissues, and enhanced anti-cancer activity comparing to native IL-15 in vivo [46]. N-803 has been reported to enhance ADCC activity mediated by mAbs against a wide range of human carcinomas [47,48]. In addition, several clinical trials are evaluating the safety and efficacy of N-803 alone or in combination with conventional cancer treatments [49,50,51].

Since NEO-201 is able to mediate ADCC to kill tumor cells, in a previous study we assessed the ability of N-803 to enhance the ADCC mediated by NEO-201, using purified NK cells from healthy donors treated with N-803 as effectors against human carcinoma cells expressing high levels of NEO-201 target antigen. In this study, we observed that N-803 significantly enhanced the ADCC activity mediated by NEO-201 against NEO-201-positive carcinoma cells in a dose-dependent manner. We also demonstrated that, after incubation with N-803 for 48 h, the expression of NK markers involved in NK cells activation and cytotoxicity, including TIM-3, NKG2D, granzyme B and CD107a, was markedly increased. In addition, we also demonstrated that N-803 modulated the phenotype of human NK cells through upregulation of gene expression of NK-activating receptors, anti-apoptotic factors and factors involved in the NK cytotoxicity, as well as through the downregulation of gene expression of NK-inhibiting receptors and factors involved in NK cell exhaustion. The ability of N-803 to upregulate gene expression of factors involved NK survival, such as the anti-apoptotic proteins Bcl-2 and DUSP-4, also allowed NK cells to have a prolonged viability compared to NK cells not stimulated with N-803. These findings provide the rationale for the development of a novel immunotherapy using long-acting IL-15 agonists in combination with NEO-201 for the treatment of NEO-201-positive tumors [30].

### 6.3. NEO-201 Can Block the Interaction between CEACAM-5 on Tumor Cells and CEACAM-1 on NK Cells and Enhances NK Cell Cytotoxicity against Human Cancer Cells In Vitro

The CEACAMs are a group of cell-surface glycoproteins secreted or bound to the plasma membrane that are overexpressed in several tumors, where they are involved in cell migration, metastatic process and drug resistance. Among the CEACAM family members, CEACAM-5 (CEA) and CEACAM-6 (NCA) play significant roles in tumor progression and metastasis [52].

CEACAM-5 was initially discovered as an oncofetal antigen found to be produced by epithelial tumor cells in the digestive tract. This glycoprotein is normally repressed during differentiation in the developing digestive tract and becomes overexpressed with de-differentiation seen in cancer, including colorectal, gastric, pancreatic, non-small cell lung and breast carcinomas [53].

CEACAM-5 is also secreted in soluble form from cancer cells into the serum, and the measurement of CEACAM-5 serum levels in cancer patients is used as a parameter for the staging and follow-up of colorectal cancer [54]. High levels of CEACAM-5 preoperatively have been correlated with metastasis and an overall poor prognosis [55], while soluble CEACAM-5 has been proved to activate endothelial cells and promote angiogenesis [56]. CEACAM-5 promotes metastasis additionally by inhibiting cells that detach from the extracellular matrix-cell contacts to undergo apoptosis in a process called anoikis. CEACAM-5 directly binds to TRAIL-R2 resulting in early inactivation of caspase-8, inactivation of caspase-9 and activation of the PI3K/Akt survival pathway. This mechanism, preventing apoptosis through anoikis, allows cancer cells to detach from their primary site, travelling through the lymphatic and circulatory system to implant and initiate tumor growth at a distant site [57,58].

CEACAM-6 is overexpressed in multiple tumor types, including breast cancer and pancreatic cancer. Overexpression of CEACAM-6 has been found to be associated with progression to invasive breast cancer [59]. CEACAM-6 is also expressed on the surface of neutrophils and acts to regulate the adhesive activity of endothelial cells through modulating the adherence to endothelial-leukocyte adhesion molecule-1 (ELAM-1) on activated endothelial cells [60].

Immunotherapy with mAbs targeting CEACAM-5 or CEACAM-6 has become an area of intense interest.

For example, a phase 1 clinical trial using yttrium-90-labeled anti-CEA antibody in combination with gemcitabine in subjects with CEA positive tumors showed partial response in 1/36 patients and 4/36 subjects with stable disease and a >50% reduction in baseline CEA levels [61].

CEACAM-1 is a cell-surface protein expressed by immune cells and tumor cells, and it plays a role in inhibiting T cell function and in promoting T cell exhaustion in colorectal cancer patients [62]. Several studies have shown that binding between CEACAM-1 on NK cells and CEACAM-1 or CEACAM-5 on tumor cells inhibits NK activation signaling mediated by NKG2D. This interaction impairs NK cell activity and allows tumor cells to evade NK killing [63,64,65].

Although several mAbs against CEACAM-5 have been found to be effective in diagnosis and therapy of several types of cancers, they failed to interfere with the CEACAM-1-CEACAM-5 interaction [31]. More recently, however, Zheng et al. identified a novel CEACAM-5 mAb that significantly suppressed cell proliferation, migration and aggregation of colorectal cancer cells in vitro and in vivo either through ADCC or through blocking intercellular interaction between CEACAM-5 on cancer cells and CEACAM-1 on NK cells [66].

Since NEO-201 reacts specifically with variants of CEACAM-5 and CEACAM-6 expressed on different human carcinoma cell lines and tumor tissues, we evaluated the capacity of NEO-201 to bind to CEACAM-5 expressed on human cancer cell lines in order to determine whether NEO-201 was able to block the CEACAM-5-CEACAM-1 interaction and to restore the antitumor functionality of NK cells. Various human carcinoma cell lines, expressing NEO-201 target antigen, were used as target cells, and NK-92 cells were used as effectors. NK-92 cells were used as a model for non-ADCC and direct NK killing of tumor cells because they express high levels of CEACAM-1 and have the CD56^+^ CD16^neg^ phenotype. We observed that NEO-201 significantly enhanced the NK-92 cell cytotoxicity only against the pancreatic cell line BxPC-3, which expressed the highest percentage of CEACAM5^+^/NEO-201^+^ cells and was also CEACAM-1 negative. NEO-201 activity in restoring NK cell cytotoxicity decreased when carcinoma cell lines with a lower level of CEACAM5^+^/NEO-201^+^ cells were used as targets, suggesting that NEO-201-mediated enhancement of NK killing is directly correlated with the level of cells expressing the variant form of CEACAM-5 recognized by NEO-201 [31].

## 7. Preclinical In Vivo Studies

### 7.1. NEO-201 Can Reduce the Growth of Tumor Xenografts Alone and in Combination with Human PBMCs Effector Cells

In two different preclinical in vivo studies, we investigated the antitumor efficacy of NEO-201 in murine models bearing human tumors expressing NEO-201 target antigen.

In one model, pancreatic cancer cell line CFPAC-1 was grown as tumor xenografts in immunocompromised NU/NU nude mice. We chose this cell line due to its high expression of the NEO-201 target antigen and its high sensitivity to NEO-201-mediated ADCC. To evaluate the in vivo ADCC activity mediated by NEO-201, tumor-bearing mice were injected three times with NEO-201 followed by three injections of IL-2-activated human PBMCs to function as ADCC-mediating effector cells. This model showed that the mice group that received NEO-201 + PBMCs had a substantial reduction in tumor growth compared to control groups that received the saline + PBMCs or human IgG + PBMCs. In addition, monitoring of the body weights of the tumor-bearing mice that received NEO-201 revealed no weight reduction in any of the treatment groups, indicating that NEO-201 is capable of substantially reducing tumor growth without inducing significant toxicity in mice. Interestingly, from biodistribution studies conducted utilizing radiolabeled NEO-201 in female and male NU/NU nude mice with established CFPAC-1 xenograft tumors, we observed that NEO-201 preferentially localizes to malignant tissue that expresses its target antigen and does not accumulate in normal tissues [28]. These data confirmed observations made with IHC of tissue samples from cancer patients.

In addition, data obtained in the pancreatic murine cancer model were also confirmed in two ovarian murine models.

In one model, to mimic primary ovarian cancer, we inoculated OV90 cells into the bursal sac surrounding the mouse ovary. Then, mice were treated with NEO-201 alone or in presence of human PBMCs as effectors for ADCC. From this model we observed that the treatment with NEO-201 or NEO-201 in combination with the PBMCs, showed a trend towards tumor control.

To evaluate whether the ADCC activity mediated by NEO-201 could also prolong the survival of tumor-bearing mice, we used a second model, where we inoculated the OV90 cells into the peritoneal cavity to mimic disseminated ovarian cancer and peritoneal carcinomatosis. In this model, we observed that mice that received NEO-201 plus activated PBMCs had a longer survival compared to mice that received NEO-201 alone or PBMCs alone [29].

Overall, these mice models demonstrated in vivo activity of NEO-201 against human cancers that specifically express its antibody target.

### 7.2. Evaluation of the Pharmacokinetics and Toxicity of NEO-201 in Non-Human Primates

The pharmacokinetics and toxicity associated with NEO-201 was determined in cynomolgus monkeys. The results from the serum chemistry, urinalysis, or coagulation tests demonstrated that there were no significant changes from baseline (pre-NEO-201 injection) through day 15 post treatment of NEO-201. We observed significant changes in neutrophil counts relative to the baseline. The decrease in neutrophil counts was a transient phenomenon in most of the animals. The neutrophil counts were recovered by day 15 [28].

## 8. Clinical Trials

**Figure 3:**
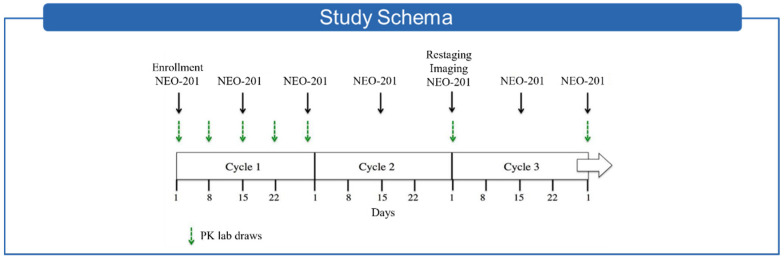
Study schema of the first in human clinical trial using NEO-201.

A first in human clinical trial using NEO-201 has completed accrual and is currently undergoing data analysis. This study accrued patients with metastatic colon cancer, pancreatic cancer, breast cancer, NSCLC, and mucinous ovarian cancer who were no longer eligible for standard therapy. As depicted in the Study Schema below (Figure 3), NEO-201 was administered intravenously on an every-2-weeks schedule [67].

This was an open label, first-in-human standard 3 + 3 phase 1 dose escalation design to determine the MTD and RP2D of the monoclonal antibody NEO-201 in adults with refractory or recurrent advanced solid tumors, a majority of whom tested positive for the NEO-201 antigen in IHC laboratory studies. Subjects were given NEO-201 intravenously every two weeks for two doses (1 cycle = 28 days) in groups of 3–6 subjects at doses of 1 mg/kg, 1.5 mg/kg, 2 mg/kg.

## 9. Conclusions

NEO-201 was found to bind to a variety of human carcinoma cell lines in vitro. In addition, IHC analysis of different human tumor tissues confirmed that NEO-201 binds specifically to tumor cells expressing a tumor-associated variant of CEACAM-5 and CEACAM-6, while it does not react against healthy tissue.

CEACAMs proteins, such as CEACAM-5 and CEACAM-6, are overexpressed in several tumors where they are involved in cell migration, metastatic process, and drug resistance. The fact that NEO-201 recognizes tumor-associated variants of CEACAM-5 and CEACAM-6 could be due to the post-translational modifications (i.e., glycosylation) occurring during the process of carcinogenesis.

The data presented here demonstrate that NEO-201 has several mechanisms of action. NEO-201 can exert a significant anti-tumor activity not only by inducing NK mediated ADCC or CDC, but also enhancing direct NK killing against CEACAM5^+^/NEO-201^+^ human carcinoma cells [28,29,30,31]. This mechanism suggests that NEO-201 can block the interaction between CEACAM-5 on tumor cells and CEACAM-1 on NK cells to reverse CEACAM-1-dependent inhibition of NK cytotoxicity [31]. We also observed that NEO-201 can bind to human regulatory T cells in human PBMCs from healthy donors [32].

In addition, we also reported previously that NEO-201 reduced the growth of human pancreatic and ovarian tumor xenografts in mice and demonstrated safety/tolerability in non-human primates, with transient neutropenia as the only adverse effect observed [28,29]. The grade of neutropenia will be investigated in cancer patient analysis from the first in human clinical trial using NEO-201 conducted at the NCI. One limitation of neutropenia in cancer patients is the increased risk of infection. To overcome this issue, adequate countermeasures, such as drugs to mitigate the duration of neutropenia or the prompt use of antibiotics, will be taken in consideration. The observed depletion of neutrophils in nonhuman primates suggests that the NEO-201 target antigen is expressed on these immune cells. This phenomenon directed us to evaluate the reactivity of NEO-201 towards human blood malignancies. Our preclinical data showed that NEO-201 was able to bind to human AML and MM cell lines. We also observed that NEO-201 was able to kill the AML cell line HL60 via ADCC [40].

These findings suggest a potential role of NEO-201 as a therapeutic agent for treating AML as well as MM. Taken together these data suggest that NEO-201 has antitumor activity and limited off-target activity, thus serving as a strong rationale for the first in human clinical trial using NEO-201 in patients with metastatic colon cancer, pancreatic cancer, breast cancer, NSCLC, and mucinous ovarian cancer, who failed standard therapy [67].

**Figure 4:**
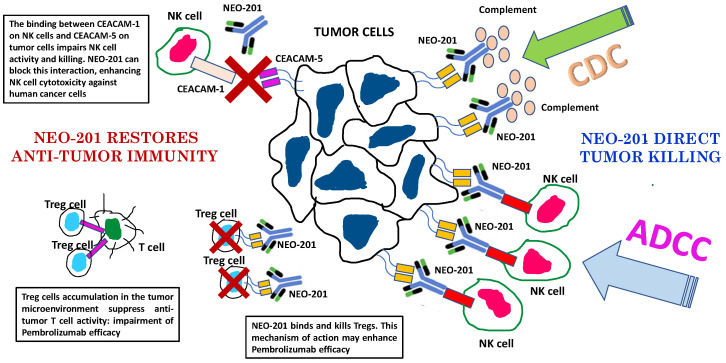
Mechanisms of action of NEO-201 and rationale to combination therapy with pembrolizumab. ADCC: antibody-dependent cell-mediated cytotoxicity. CDC: complement dependent cytotoxicity. Although the majority of cancer patients treated with PD-1/PD-L1 blockade monotherapies do not achieve objective responses, when responses are observed, most tumor regressions are partial rather than complete [68]. The low response rates and resistance to PD-1/PD-L1 blockade could be due to the activity of regulatory T cells in the TME [69,70,71].

Monoclonal antibody-based immunotherapies have been shown to be relatively effective as anticancer therapies. While mAb monotherapy has demonstrated success in some cancer patients, combination therapy with standard chemotherapy, radiation, tyrosine kinase inhibitor, immune checkpoint inhibitors and other cellular therapies may enhance therapeutic responsiveness and reducing adverse side effects. For example, the fact that NEO-201 recognizes human regulatory T cells, as well as its direct activity to kill tumor cells, mediating ADCC and CDC, can serve as a rationale for combination of NEO-201 with checkpoint inhibitors such as pembrolizumab (Figure 4).

Depletion of highly suppressive regulatory T cells in tumor tissues could be an effective strategy to prevent hyperprogressive disease when anti-PD-1 mAb treatment is administered as cancer immunotherapy. These data support the rationale for clinical trials using NEO-201 in combination with ICIs targeting the PD-1/PD-L1 pathway to improve patient outcomes in a wide variety of cancers.

## Figures and Tables

**Table 1:** NEO-201 IHC of tumor tissue microarrays.

Cancer Tissue Type	Positive/Spot	Cancer Tissue Type	Positive/Tpot
Brain:astrocytoma	0/2	Lung papillary carcinoma	0/2
Brain:choroid plexus papilloma	0/2	Lung metastatic papillary carcinoma	2/2
Esophagus squamous cell carcinoma (SCC)	4/4	Liver cholangiocarcinoma	0/2
Larynx SCC	0/2	Liver metastatic lung large cell carcinoma	0/2
Thymus atypical carcinoma	0/2	Hepatocellular carcinoma (HCC)	0/4
Thyroid papillary carcinoma	0/4	Renal cell carcinoma (RCC)	0/4
Thyroid invasive follicular carcinoma	0/2	Ovary germ cell carcinoma	1/1
Thyroid follicular carcinoma	0/2	Ovary serous carcinoma	2/2
Breast infiltrating ductal carcinoma	0/4	Ovary clear cell carcinoma	0/2
Stomach adenocarcinoma	3/4	Ovary mucinous carcinoma	0/2
Pancreas papillary mucinous carcinoma	2/2	Cervical SCC metaplasia	0/2
Pancreas adenocarcinoma	0/1	Cervical invasive SCC	2/2
Tongue SCC	2/4	Testis seminoma	0/4
Non-small cell lung cancer (NSCLC)	0/2	Colon adenocarcinoma	4/4
Lung adenocarcinoma	2/2	Rectum adenocarcinoma	3/4
Lung SCC	1/2	Skin SCC	0/4
Lung large cell carcinoma	2/2		

**Table 2:** NEO-201 IHC profile: results from lung cancer microarray.

Lung Cancer Type	Positive#/Total Case (% Reactivity)
Lung adenocarcinoma	27/34 (79.4%)
Lung Squamous Cell Cancer	18/34 (52.9%)
NOS non-small cell lung cancer	0/4 (0%)
Bronchi alveolar Cancer	1/2 (50%)
Large cell neuroendocrine Cancer	0/1 (0%)
Lung Cancer (Total)	46/75 (61.3%)

NOS = not otherwise specified.
